# Left ventricular hemodynamic forces as a marker of mechanical dyssynchrony in heart failure patients with left bundle branch block

**DOI:** 10.1038/s41598-017-03089-x

**Published:** 2017-06-07

**Authors:** Jonatan Eriksson, Jakub Zajac, Urban Alehagen, Ann F. Bolger, Tino Ebbers, Carl-Johan Carlhäll

**Affiliations:** 1 Division of Cardiovascular Medicine, Department of Medical and Health Sciences, Linköping University, Linköping, Sweden; 2 Center for Medical Image Science and Visualization (CMIV), Linköping University, Linköping, Sweden; 3 Department of Cardiology, Department of Medical and Health Sciences, Linköping University, Linköping, Sweden; 4 Department of Medicine, University of California, San Francisco, California USA; 5 Division of Media and Information Technology, Department of Science and Technology/Swedish e-Science Research Centre (SeRC), Linköping University, Linköping, Sweden; 6 Department of Clinical Physiology, Department of Medical and Health Sciences, Linköping University, Linköping, Sweden

## Abstract

Left bundle branch block (LBBB) causes left ventricular (LV) dyssynchrony which is often associated with heart failure. A significant proportion of heart failure patients do not demonstrate clinical improvement despite cardiac resynchronization therapy (CRT). How LBBB-related effects on LV diastolic function may contribute to those therapeutic failures has not been clarified. We hypothesized that LV hemodynamic forces calculated from 4D flow MRI could serve as a marker of diastolic mechanical dyssynchrony in LBBB hearts. MRI data were acquired in heart failure patients with LBBB or matched patients without LBBB. LV pressure gradients were calculated from the Navier-Stokes equations. Integration of the pressure gradients over the LV volume rendered the hemodynamic forces. The findings demonstrate that the LV filling forces are more orthogonal to the main LV flow direction in heart failure patients with LBBB compared to those without LBBB during early but not late diastole. The greater the conduction abnormality the greater the discordance of LV filling force with the predominant LV flow direction (r^2^ = 0.49). Such unique flow-specific measures of mechanical dyssynchrony may serve as an additional tool for considering the risks imposed by conduction abnormalities in heart failure patients and prove to be useful in predicting response to CRT.

## Introduction

Heart failure is a common clinical problem and adverse cardiac remodeling is a key component in the deterioration of cardiac function, in the downward spiral leading towards heart failure. Increased myocardial wall stress during diastole contributes to the development and progression of adverse cardiac remodeling^1^. Around 25 percent of patients with congestive heart failure may have Left Bundle Branch Block (LBBB)^2^. LBBB causes dyssynchronous LV contraction and relaxation, and contributes to impaired left ventricular (LV) performance. The majority of studies have investigated the impact of LBBB on systolic LV function. Dyssynchronous and delayed relaxation may have important impact on LV filling properties and diastolic function as well.

Time-resolved three-directional three-dimensional velocity (4D flow) data, acquired by magnetic resonance imaging (MRI), provides important information regarding intracardiac blood flow and cardiac function^3–6^. Previous studies have shown that the distributions of different flow components comprising the end diastolic volume (EDV) of LV may be markers of cardiac function^7–11^. Another approach to assess intracardiac blood flow has been calculation of the kinetic energy of the blood^5, 7, 8^. In this approach, the directionality of the blood flow is lost. Furthermore, from the velocity field and by the use of governing equations, pressure gradients and relative pressure fields can be calculated^12–15^. By integration of the pressure gradients throughout the LV blood pool, a vector is obtained describing the total force between the LV blood and the surrounding myocardial walls: the LV hemodynamic force.

The hemodynamic force concept is based upon Newton’s third law (the action-reaction law). According to this law, the accelerating and decelerating blood exerts a force on the surrounding myocardial walls, while the myocardial walls exert a force on the blood. These forces are equal in size, but opposite in direction. As blood flows from the left atrium into the LV it will augment the pressure in the LV, resulting in deceleration of the blood. The blood will have higher velocity in the basal part of the LV and decelerate as it progresses towards the apex. This means that the pressure gradient will be positive along the flow direction (i.e. the pressure is increasing along the flow direction), hence, the hemodynamic force, the force the blood exerts on the myocardial wall, will be directed towards the apex. During systole the velocity is increased from apex towards the LVOT, the pressure gradient will be negative from apex to LVOT, i.e. the pressure will decrease in the flow direction and the hemodynamic force will be directed in the opposite direction. Note that the myocardium will exert a force on the blood that is equal in size, but opposite in direction.

The concept of LV hemodynamic force has been known for decades^16^, but was only recently computed from 2D LV flow fields by Pedrizetti *et al*., using echocardiographic particle image velocimetry (echo-PIV)^17, 18^. Using this technique, the directional distribution of hemodynamic forces in the LV of heart failure patients with cardiac resynchronization therapy (CRT) has been investigated^19^. In this study patients with dilated cardiomyopathy that had undergone CRT implantation were examined by echocardiography before and after a temporary deactivation of the CRT. The results showed that the orientation of flow momentum changed from along the LV long axis direction to along the short axis direction during deactivation.

We extended this approach for 3D flow field obtained from 4D flow MRI^20^. In a recent study, we hypothesized that in a healthy heart the hemodynamic force should be directed concordant with the main flow direction, apex-to-base, and that changes in this pattern would reflect abnormal LV function^20^. Therefore, we examined the relative distribution of the hemodynamic force along the long (apex-to-base) axis and that acting in the short (anteroseptal-to-inferolateral) axis during diastolic filling. In a heterogeneous group of dilated cardiomyopathy patients we found that the ratio of forces acting in the long- and short-directions was different from normal, especially during early filling, with a relative increase in the hemodynamic forces acting orthogonal to the main flow axis^20^.

In this work we sought to investigate the impact of LBBB-related dyssynchronous LV relaxation on global LV diastolic hemodynamics and function. We hypothesized that in heart failure patients with LBBB, the LV hemodynamic filling forces would be more discordant with the axis of the early inflow, when disordered repolarization would have its greatest impact on filling, compared to matched patients without LBBB, and could therefore serve as a marker of diastolic mechanical dyssynchrony.

## Results

MRI data were successfully acquired in heart failure patients with LBBB and matched patients without LBBB (from hereby referred to as the non-LBBB group). As expected, the QRS duration was significantly longer in LBBB versus non-LBBB patients (154 (64) ms vs 106 (24) ms, P < 0.0001). There were no significant differences between the groups with respect to demographic and clinical parameters, such as age, body size, heart rate, blood pressure, LVEDV-index, and LV ejection fraction (Table 1). Notably, there was no significant difference in LV sphericity index between the groups (0.60 ± 0.08 vs 0.65 ± 0.10, P = 0.329). The LV septal to lateral wall delay was 57.6 ± 62.0 ms in LBBB patients and −4.4 ± 54.9 ms in non-LBBB patients (P = 0.039).

**Table 1:** Demographic and clinical parameters.

Parameter	LBBB group	Non-LBBB group	P-value
Age (years)	63 ± 11	59 ± 14	0.5234
Gender (m:f)	8:1	8:1	N/A
Length (cm)	174 ± 9	173 ± 7	0.8075
Weight (kg)	83 ± 17	84 ± 14	0.8324
BSA (m^2^)	2 ± 0.2	2 ± 0.2	0.8646
HR (bpm)	68 ± 12	69 ± 10	0.8972
BP Syst (mmHg)	137 ± 15	133 ± 23	0.6126
BP Diast (mmHg)	77 ± 6	84 ± 18	0.2819
Etiology (ICMP:IDCMP)	3:6	3:6	N/A
LVEDV (ml)	274 ± 92	247 ± 71	0.4933
LVEDV index (ml/m^2^)	138 ± 48	123 ± 30	0.4097
LV Sphericity index	0.60 ± 0.0810	0.65 ± 0.1035	0.3291
LVEF (%)	32 ± 8	34 ± 7	0.5593
QRS-time (ms)	154 (64)	106 (24)	<0.0001

M, male; F, female; BSA, Body Surface Area; HR, Heart Rate; BP, Blood Pressure; LV, Left Ventricle; EDV, End-diastolic Volume; EF, Ejection Fraction.

LV pressure gradients were calculated from the Navier-Stokes equations. Integration of the pressure gradients over the LV volume rendered the hemodynamic force. The hemodynamic forces showed a peak directed towards the atrium at the onset of both early and late diastolic filling phases in all patients except one without LBBB. Additional peaks in the hemodynamic forces directed towards the apex could be observed after early diastolic filling in all patients except one with LBBB and following late diastolic filling in all 18 patients.

**Figure 1:**
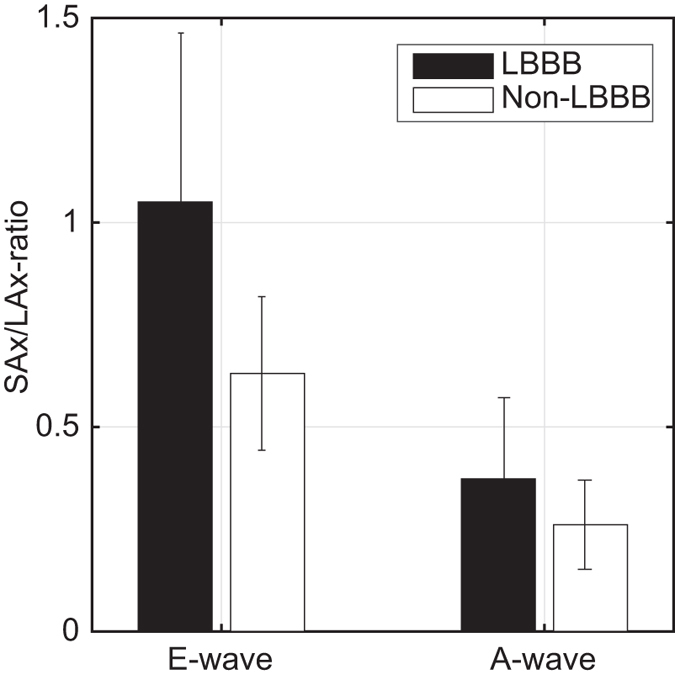
Mean ± std of the “SAx-max force/LAx-max force”-ratio during early diastolic filling (E-wave) and late diastolic filling (A-wave) respectively for the LBBB-patients (black bars) and non LBBB- patients (white bars). During E-wave the ratio was significantly larger for the LBBB patients compared to non-LBBB patients (P-value = 0.020), while there was no significant intergroup difference during A-wave (P-value = 0.185).

The SAx-max force/LAx-max force (from hereon called the SAx/LAx-ratio) was defined as the ratio between the maximum force along the anteroseptal-to-inferolateral axis in the SAx-view and the maximum force along the apex-to-base axis in the LAx three-chamber view. This ratio, reflecting the deviation of the LV hemodynamic forces from the main LV flow direction, was significantly larger in the LBBB group compared to the non-LBBB for the complete diastolic phase (0.59 ± 0.28 vs 0.35 ± 0.16, P = 0.038). During early diastolic filling, the SAx/LAx-ratio was significantly larger in the LBBB group compared to the non-LBBB (1.05 ± 0.41 vs 0.63 ± 0.19, P = 0.020) (Fig. 1). Such intergroup difference was not observed during late diastolic filling (0.37 ± 0.2 vs 0.26 ± 0.11, P = 0.185) (see also Supplementary Figures 1 and 2). The mean difference between the time points when max SAx and max LAx forces occurred during early filling was 91 ± 46 ms and during late filling 48 ± 35 ms.

In order the further evaluate the time profile of the hemodynamic forces, the root mean square (rms) of the force-ratio was computed over all diastolic phases. The SAx/LAx-ratio_rms_ was larger in the LBBB-group compared to the non-LBBB group for full diastole (0.61 ± 0.21 vs. 0.40 ± 0.09, P = 0.011), showed a trend to be larger at early filling (0.90 ± 0.34 vs. 0.62 ± 0.16, P = 0.054) but showed no inter-group difference at late filling (0.33 ± 0.15 vs. 0.27 ± 0.11, P = 0.3804).

**Figure 2:**
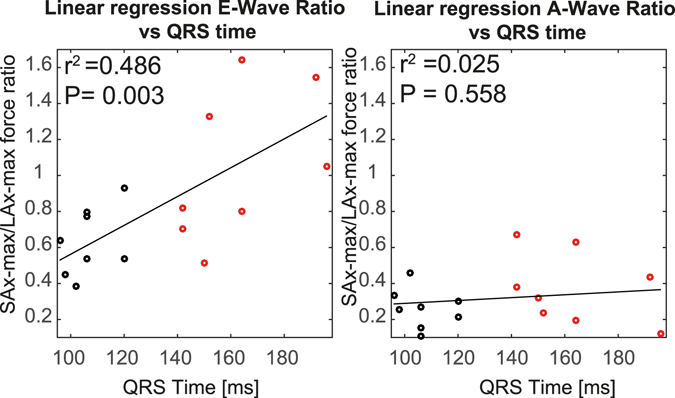
Regression analysis of the “SAx-max force/LAx-max force”-ratio versus the QRS duration for (left panel) E-wave and (right panel) A-wave. The black solid line is the best fit of a line to the data; red circles represent the non-LBBB patients and black circles the LBBB patients.

During early diastolic filling there was a moderate to strong correlation between the SAx/LAx-ratio and QRS duration (r^2^ = 0.4857, P = 0.003), while there was no correlation between these two parameters during late diastolic filling (r^2^ = 0.0251, P = 0.558), using linear regression analysis (Fig. 2). Also during early filling there was a modest to moderate correlation between the SAx/LAx-ratio and septal-lateral delay (r^2^ = 0.2418, P = 0.053), whereas no correlation was observed during late filling (r^2^ = 0.0286, P = 0.531).

## Discussion

In the current study we utilized a recently presented MRI-based method to calculate and analyze the LV 4D hemodynamic filling forces in a group of heart failure patients with LBBB and compare the results to patients without LBBB but with similar age, gender, heart rate and LV characteristics. The SAx/LAx-ratio, a measure of the deviation of the LV hemodynamic forces from the main flow direction, was higher during the early diastolic filling phase in the patients with dyssynchronous LV relaxation (LBBB patients) compared to the matched patients (non-LBBB), while no intergroup difference was observed during late diastolic filling. The ratio computed with root mean square values for the force components in the LAx and SAx showed the same trend during diastole as the ratio of max values. Moreover, we found that the SAx/LAx-ratio and QRS duration correlated during early diastole but not during late diastole.

As recently observed in normal and myopathic LVs^20^, the LV hemodynamic forces in heart failure patients in this study also showed a peak directed towards the atrium at onset early and late filling, due to the acceleration of the blood, and towards the apex following early and late filling, due to the deceleration of the blood in the apical region. In the same recent study, a relatively homogenous pattern of hemodynamic filling forces was demonstrated in normal LVs, where the forces were directed concordant with the main flow directions, compared to a considerably more heterogeneous pattern in myopathic LVs, where the forces were to a greater extent orthogonal to the main flow direction^20^. Arvidsson *et al*.^21^ showed that also elite athletes’ LV forces concordant with the main flow direction dominated throughout the cardiac cycle and there were no differences between athletes and healthy volunteers. In addition, two case patients with ventricular dyssynchrony were examined and found to have different force patterns with the LV forces orthogonal to the main flow direction equal to the forces along the main flow direction. In the present study, the intriguing findings from the SAx/LAx-ratios propose that the forces are orthogonal to the main flow direction to a greater extent in the patient group with LBBB compared to the patient group without LBBB during the early diastolic filling phase.

Hence, the current heart failure patients with LBBB demonstrated altered LV blood flow dynamics due to changes in the electrical activation of the ventricular myocardium. In an interesting echo-PIV study by Pedrizzetti *et al*.^19^ similar findings were shown in a patient group with CRT where the orientation of blood flow momentum was altered and became more orthogonal to the main LV flow direction when the CRT was deactivated. Although the aim of CRT is to resynchronize intra- and inter-ventricular motion patterns, however, active pacing does not achieve normal electrical activation through the conduction system. Furthermore, deactivation of CRT for a few minutes probably does not completely reflect the underlying ventricular dyssynchrony patterns. Therefore, the findings of this ultrasound study cannot be directly compared to the findings of the present study.

In the current study, there was no difference in the sphericity index between the two patient groups, implying that the increased SAx/LAx-ratio in the LBBB group is not a result of the altered geometry of the failing ventricles. Altered LV intracavitary flow patterns have previous been demonstrated in enlarged and dysfunctional ventricles^22–24^, and it is reasonable to believe that LBBB related alterations in the transit of blood through the chamber may contribute to the difference in force direction between the groups.

The present study puts emphasis on a mechanism that may be reflected by the SAx/LAx-ratio. In LBBB patients with dyssynchronous LV electrical and mechanical function, RV diastolic filling precedes LV diastolic filling due to the delayed LV relaxation^25^. This delay can lead to a transeptal pressure gradient that allows the interventricular septum to bulge into the LV cavity during early diastole^26^. Combined with the delayed outward motion of the opposing LV lateral wall, this distortion could influence flow patterns and contribute to the relative increase of the maximal force in SAx plane during early filling.

Interestingly, the moderate to strong relationship between the QRS duration and the SAx/LAx-ratio suggests that the greater the conduction abnormality the larger the relative increase in maximal force in the SAx plane.

The absence of intergroup difference in SAx/LAx-ratios at late diastole could be explained by that phase’s distinct filling mechanisms that incorporate atrial contract and annular recoil. Hence, late filling is less affected by dyssynchronous LV myocardial relaxation and motion.

The current findings propose new blood flow based aspects of mechanical dyssynchrony that may prove to contribute to the development and progression of pathological cardiac remodeling. Altered direction and temporal progression of hemodynamic filling forces may alter the pattern of global myocardial wall stress during diastole, which in turn could have impact on the remodeling process^1^. Thus, a LBBB related increase in SAx/LAx-ratio, with relatively higher global force that is discordant with the predominant LV flow direction, could contribute to the development and progression of pathological cardiac remodeling. Interestingly, Pedrizzetti and colleagues showed in their work that the deviation of blood flow momentum from the predominant LV flow direction during deactivation of CRT, correlated with the degree of reversed LV adverse remodeling (reduction in LV volumes) following CRT implantation^19^.

Potential areas of future application and clinical utility include cardiac resynchronization therapy (CRT). Despite the documented benefits of CRT in many patients, a significant proportion of patients undergoing this therapy do not demonstrate improvement^27^. This is an essential clinical issue, since the number of potential patients is enormous, and the cost per device is high. Specific characteristics of 4D LV hemodynamic filling forces might prove useful in predicting response to CRT and also in future optimization of CRT settings.

It is reasonable to expect that LV hemodynamic forces would also be sensitive to conditions associated with altered LV inflow direction, such as with mitral valve dysfunction or prosthetic replacement, or in the setting of significant regional wall motion abnormalities due to myocardial infarction. The impact of these conditions on hemodynamic forces will be pursued in future works.

The SAx/LAx-ratio was computed using the maximum values in SAx and LAx planes, which potentially may be sensitive to noise and artifacts. In order to provide a temporally global measurement less sensitive to noise, the root mean square (rms) of the SAx/LAx-ratio was computed over all diastolic phases, including E-wave as well as A-wave. The rms of the force ratio in the LAx and SAx (SAx/LAx-ratio_rms_) showed the same trend throughout diastole as the force-ratio with the max values (SAx/LAx-ratio): this finding suggests that both values are valid.

In this pathophysiological study, utilizing a cutting edge cardiac imaging methodology, the findings relate only to a relatively small number of heart failure patients. However, great effort was put into matching the two patient groups to each other; for every heart failure patient with LBBB included, another heart failure patient without LBBB but with the same etiology was matched according to age, gender, heart rate and LV characteristics, in the priority order defined in the methods section, was identified.

The current study included three LBBB patients with ICMP and three matched non-LBBB patients with ICMP. As LV myocardial hypokinesia or akinesia due to ischemic heart disease may influence intraventricular flow and hemodynamic forces, this type of LV wall motion abnormality was assessed in these ICMP patients to assure that each LBBB patient were matched to a non-LBBB patient with similar location and extent of the LV myocardial hypokinesia or akinesia.

One patient had to be excluded during the analysis of the separate E- and A-wave ratios because the E-wave could not be defined. Possible reasons are a combination of a very short diastasis (underlying conduction abnormality and relative high heart rate) and the temporal resolution of the data in this study.

In the computation of the hemodynamic forces, the viscous terms in Navier-Stokes equations might be underestimated. They are merely important when assessing parameters in the shear layer close to the walls, however, and therefore should be a minor determinant of the forces examined in the present study. Noise and other artifacts are known to affect the spatial gradients in the Navier-Stokes equations. The effect on the hemodynamic forces is expected to be small, however, as these are derived by integrating the pressure gradients over the entire LV volume.

The temporal resolution of the velocity data was 52.8 ms. While better temporal resolution would be preferable, particularly in patients with higher heart rates, this level represents an acceptable tradeoff between acquisition time and temporal resolution. Further, one of the matching parameters between the patient groups is heart rate, so any effects caused by temporal resolution should be present in both groups and not impact the comparisons.

Analysis of LV hemodynamic forces is a new and comprehensive approach to visualize and quantify the 4D LV intracavitary blood volume. LV filling forces are orthogonal to the main flow direction to a greater extent in patients with LBBB compared to patients without LBBB during early but not during late diastole. The greater the conduction abnormality the greater the discordance of LV filling force with the predominant LV flow direction. Such unique flow-specific measures of mechanical dyssynchrony may serve as an additional tool for considering the risks imposed by conduction abnormalities in heart failure patients and prove to be useful in predicting response to CRT.

## Methods

### Study population

Eighteen heart failure patients with ischemic cardiomyopathy (ICMP) or idiopathic dilated cardiomyopathy (IDCMP) were divided into two different groups; 1) patients with LBBB, and 2) patients matched according to gender, age, heart rate, and parameters of LV characteristics without LBBB. Demographic and clinical data for the subjects are presented in Table 1.

Inclusion criteria for 1) ICMP patients: diagnosed with ischemic heart disease based on myocardial perfusion scintigraphy tests and/or MRI evidence of myocardial infarction using late gadolinium enhancement; at least mild to moderate systolic LV dysfunction (ejection fraction <45%); 2) IDCMP patients: absence of other (secondary) etiology of DCMP; at least mild to moderate systolic LV dysfunction (ejection fraction <45%); at least mild LV dilatation; 3) LBBB patients: QRS duration >120 ms and typical LBBB pattern on the QRS complex and T wave. Exclusion criteria for all patients were contraindication for MRI examination, significantly irregular cardiac rhythm, heart rate <40 or >100 bpm, more than mild valvular disorder or discrepancy of >15% in the MRI-based estimates of LV inflow and outflow.

The patients were enrolled from outpatients at the Department of Cardiology, Linköping University Hospital.

All subjects gave written informed consent prior to participation in the study. The study was performed in accordance with the Helsinki declaration and the Regional Ethical Review Board in Linköping. The Regional Ethical Review Board in Linköping also approved the study protocols.

### Data acquisition and post processing

All subjects underwent a cardiac MRI examination using a clinical 3T MRI scanner (Philips Ingenia, Philips Medical Systems, Best, the Netherlands).

During the MRI examination, morphological two-, three- and four-chamber long-axis (LAx) images and a stack of short axis (SAx) images were acquired. Time-resolved three-directional velocity (4D flow) data were acquired in a volume encompassing the heart, by the use of a turbo field echo sequence with interleaved bipolar flow encoding gradients. The data were acquired during free-breathing using retrospective navigator gating. The field of view was set in a sagittal position and the number of slices in the slab was adjusted to encompass the heart of each study subject. General acquisition parameters were: spatial resolution, 2.8 mm isotropic; flip angle, 10° (or 5°, in five of the subjects we did not administer contrast agent); repetition time, 4.4 ms, echo time, 2.6 ms; velocity encoding, 120 cm/s. A k-space segmentation factor of 3 was applied and parallel imaging with sensitivity encoding (SENSE) with a speed-up factor of 3 was applied and elliptical k-space acquisition was used. These settings gave a temporal resolution of 52.8 ms. The patients were scanned in supine position. Mean total scan duration for the 4D flow acquisition was 16.7 ± 6.9 minutes. All patients (with the exception of five) were given a gadolinium contrast agent (Magnevist, Bayer Schering Pharma AG) prior to the data acquisition for a late gadolinium enhancement study.

After data acquisition the 4D flow data were reconstructed into 40 time frames on the scanner and exported to an offline station for post processing and analysis. The data was post processed by the use of in-house developed software written in Matlab (The Mathworks Inc., Natick, Massachusetts, USA). The 4D flow data were corrected for background errors by the use of a 4^th^ order polynomial fitted to the static tissue in the thorax^28^ and for phase wraps by the use of a temporal algorithm^29^.

### Data analysis

**Figure 3:**
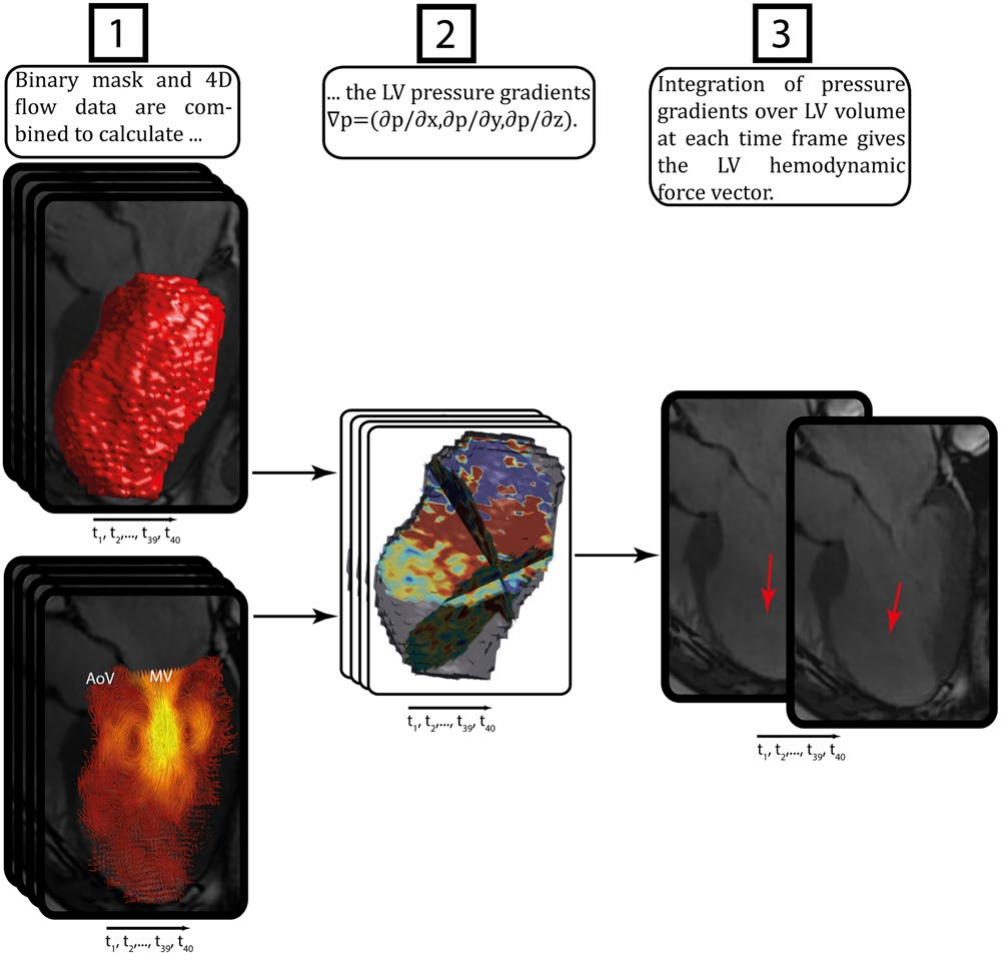
Schematic overview of the analysis method: (1) a binary mask is generated from the segmentation of the bSSFP short axis images and superimposed on magnitude data from the 4D flow scan, and adjusted in order to avoid possible mismatch problems between bSSFP-images and flow data, (2) the LV pressure gradients ∇p = (∂p/∂x, ∂p/∂y, ∂p/∂z) are calculated by using the velocity data and binary mask, (3) integration of pressure gradients over the LV volume at each time frame gives the LV hemodynamic force vector.

The LV was segmented at all time frames from SAx stack, with aid from the LAx images in order to determine the most basal slice, by the use of semi-automatic segmentation software (Segment, Medviso AB, Lund, Sweden). The segmentation was resampled to the same number of time frames as the 4D flow data. The segmentation was manually corrected for possible mismatches due to patient motion assisted by superimposition of the segmentation on the magnitude data of the 4D flow MRI in the SAx orientation. The corrected segmentation was then used to create a binary mask containing the LV lumen. The binary mask was used as boundary conditions to calculate the spatial and temporal gradients in the Navier-Stokes equation (the body forces, f, were excluded): $\nabla p=-\rho \frac{\partial v}{\partial t}-\rho v\nabla v+\mu {\nabla }^{2}v$, where *v* [m/s] is the velocity, ρ the density (1060 [kg/m^3^]) and μ the viscosity (0.004 [Ns/m^2^]) of blood. By integrating the pressure gradients, $\nabla {\rm{p}}$, over the LV volume at every time frame, the 3D hemodynamic force vector was calculated for every time frame of the cardiac cycle (Fig. 3).

**Figure 4:**
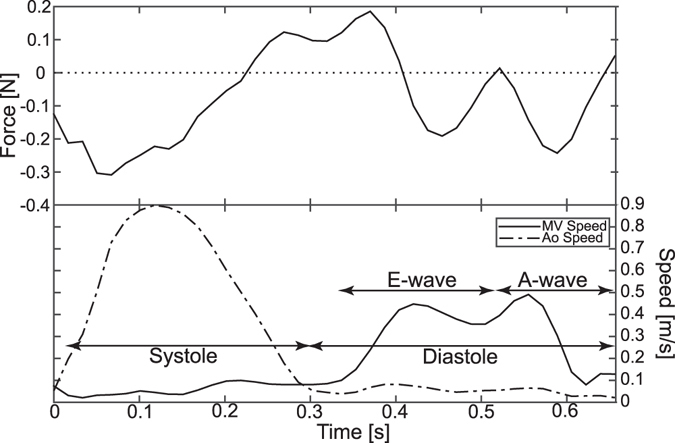
(Top panel) Hemodynamic force [N] where a direction of the vector is considered positive if it is mainly directed towards atrium and negative if it is mainly directed towards apex. (Bottom panel) Speed data [m/s] extracted from the 4D flow data at points near the mitral valve (MV) orifice and the aortic valve (AoV) orifice. These speed curves in combination with streamline visualizations are used to divide the cardiac cycle into systole and diastole, the latter with subphases of early (E) and late (A) filling. The x-axis shows time in seconds from R-peak.

Speed data were extracted from two points set in the 4D flow data, at the atrioventricular plane near the mitral valve orifice and near the aortic valve orifice, and plotted over time (Fig. 4). These data, in combination with visualizations of the blood flow made by emitting streamlines for 25 ms from a LAx three-chamber image, was used to separate the cardiac cycle into different phases: systole and diastole. Further, diastole was divided into early (E-wave) and late (A-wave) filling.

**Figure 5:**
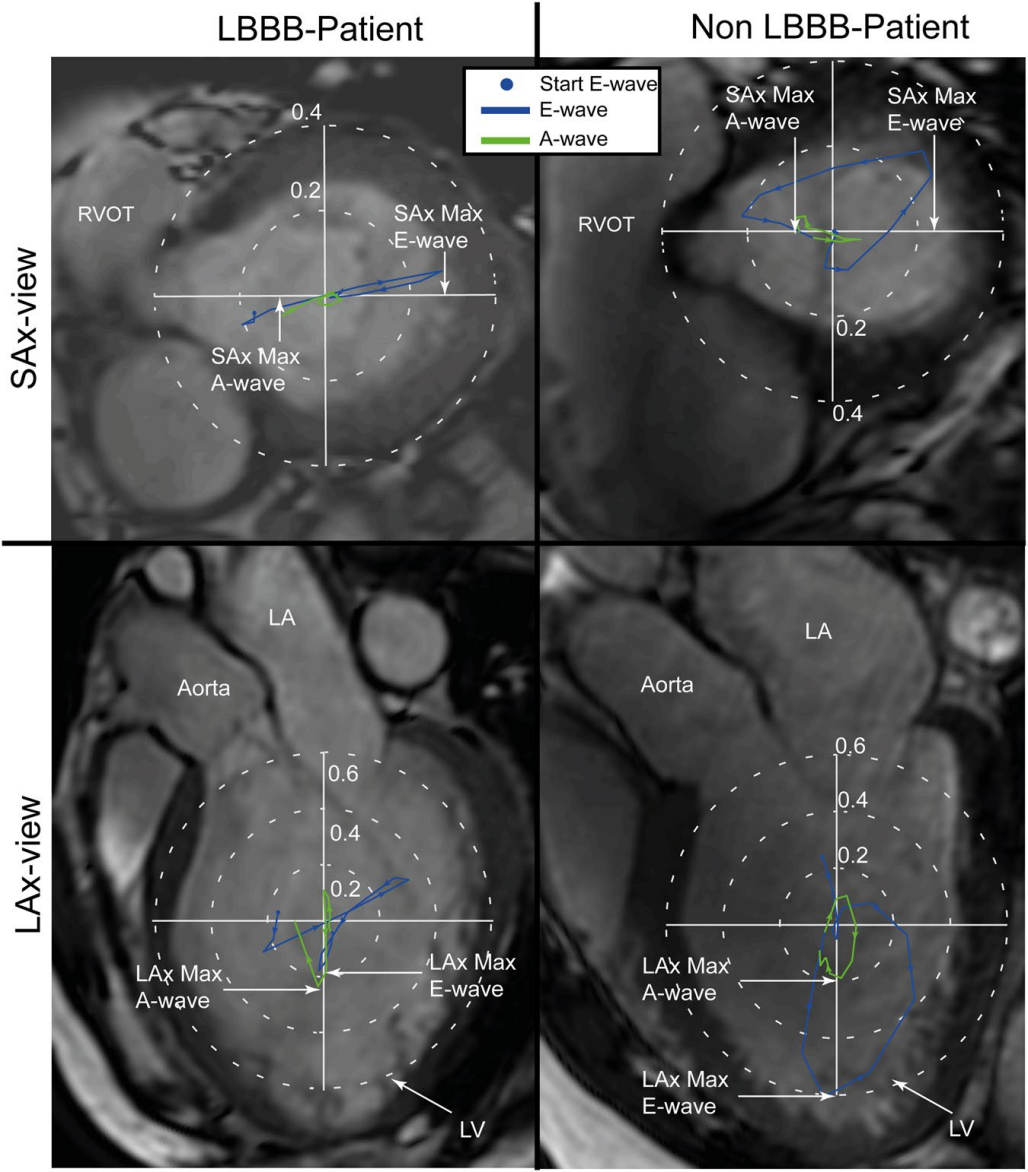
Hemodynamic forces [N] in a LBBB patient (left panels) and in a non-LBBB patient (right panels) projected onto a SAx-image (top) and LAx-image (bottom). The hemodynamic force plots are colored according to diastolic phases, E-wave (blue) and A-wave (green). The “SAx-max force/LAx-max force”-ratio was defined as the ratio between the maximum force along the anteroseptal-to-inferolateral axis in the SAx-view and the maximum force along the apex-to-base axis in the LAx three-chamber view. E-wave, early diastolic filling; A-wave, late diastolic filling; LA, left atrium; LV, left ventricle.

In order to assess the heterogeneity of the force directions the “SAx/LAx-ratio” was calculated (Fig. 5). This ratio was defined as the maximum force along the anteroseptal-to-inferolateral axis in the SAx view divided by the maximum force along the apex-to-base axis in the LAx three-chamber view. Forces along the anteroseptal-to-inferolateral axis in the SAx view can be influenced by the delayed outward motion of LV lateral wall and also by the direction of the LV inflow (generally towards the inferolateral wall) and outflow (generally towards anteroseptal wall)^8^. The SAx/LAx-ratio was calculated over the whole diastolic phase, and also over the duration of the early (E-wave) and late (A-wave) filling phases separately. The maximum forces in the SAx view and the LAx view were not necessarily extracted at the same time frame, e.g. the E-wave SAx/LAx-ratio was calculated by finding the maximum forces in the LAx and SAx-directions, each at any time during the early diastolic phase.

In order to provide a temporally inclusive measurement, the rms of the SAx/Lax ratio (SAx/LAx-ratio_rms_) was the calculated over all diastolic phases.

The subjects also underwent standard electrocardiographic (ECG) and echocardiographic examinations. The electrocardiographic examination was performed by experienced nurses at the Department of Cardiology, Linkoping University Hospital, using a GE MAC 5500 HD. For inclusion criteria purposes, an experienced investigator assessed the presence of LBBB according to standard definitions: QRS duration > 120 ms and typical LBBB changes to the QRS complex and T wave^30^. The transthoracic echocardiographic examination was performed by experienced ultrasound technicians at the Department of Clinical Physiology, Linkoping University Hospital, using a Vivid E9 scanner (GE, Vingmed Ultrasound, Horten, Norway). An experienced investigator assessed the presence of left sided regurgitant or stenotic valvular disease for exclusion criteria purposes, and LV myocardial hypokinesia or akinesia in patients with ICMP, according to standard recommendations^31^.

Measurements of mechanical synchrony were performed in each patient as the time delay between the mechanical activation of the LV septal and lateral wall in a four-chamber echocardiographic view using Siemens *Syngo* Velocity Vector Imaging software version 2.00. The mechanical activation was defined as the mean time delay from the onset of the QRS complex to peak tissue velocity in both longitudinal and radial directions.

### Statistical analysis

The Lilliefors method was used to test the data for normal distribution. Inter-group comparisons were analyzed using t-tests for normally distributed data and Wilcoxon Rank Sum tests for non-normally distributed data (QRS-time). All data are presented as mean ± standard deviation or median (range). Linear regression was used to analyze the relation between SAx/LAx-ratio and QRS duration for E-wave and A-wave, and between SAx/LAx-ratio and septal-lateral delay for E-wave and A-wave. A P-value < 0.05 was considered significant. In one of the LBBB patients the E-wave could not be defined; that patient was excluded from analysis of early versus late diastolic phases.

### Data availability

The underlying magnetic resonance imaging, echocardiographic and electrocardiographic data sets are available at the Linköping University Hospital for researchers who meet the criteria for access to confidential data. The computed hemodynamic forces are available from the author upon request.

## Electronic Supplementary Information

Supplementary Informationpdf
