# Supplementary material for: Left ventricular hemodynamic forces as a marker of mechanical dyssynchrony in heart failure patients with left bundle branch block
Source: Sci Rep. 2017 Jun 7;7:2971. doi: 10.1038/s41598-017-03089-x (PMC5462838; doi:10.1038/s41598-017-03089-x)
Supplement: Supplementary file 1 — Supplementary Informationpdf [file 41598_2017_3089_MOESM1_ESM.pdf]

Left ventricular hemodynamic forces as a marker of mechanical dyssynchrony in heart failure patients with left bundle branch block

Jonatan Eriksson, PhD,[1] Jakub Zajac, MD,[1,2] Urban Alehagen, MD, PhD,[3] Ann F Bolger, MD,[1,4] Tino Ebbers, PhD,[1,2,5] Carl-Johan Carlhäll, MD, PhD,[1,2,6*]

[1]Division of Cardiovascular Medicine, Department of Medical and Health Sciences, Linköping University, Linköping, Sweden.

[2]Center for Medical Image Science and Visualization (CMIV), Linköping University, Linköping, Sweden

[3]Department of Cardiology, Department of Medical and Health Sciences, Linköping University, Linköping, Sweden

[4]Department of Medicine, University of California, San Francisco, California, USA

[5]Division of Media and Information Technology, Department of Science and Technology/Swedish e-Science Research Centre (SeRC), Linköping University, Linköping, Sweden

[6]Department of Clinical Physiology, Department of Medical and Health Sciences, Linköping University, Linköping, Sweden

***Corresponding author:**

Carl-Johan Carlhäll, MD, PhD

Division of Cardiovascular Medicine

Linköping University

SE-58183 Linköping, Sweden

E-mail: carljohan.carlhall@liu.se

Supplementary Information

Supplementary Figure 1: Hemodynamic forces [N] during diastole projected onto a short-axis (SAx)-plane. The hemodynamic force plots are colored according to diastolic phases, E-wave (blue) and A-wave (green). The x axis is aligned along the anteroseptal-to-inferolateral axis. E-wave, early diastolic filling; A-wave, late diastolic filling; LVOT, left ventricular outflow tract.

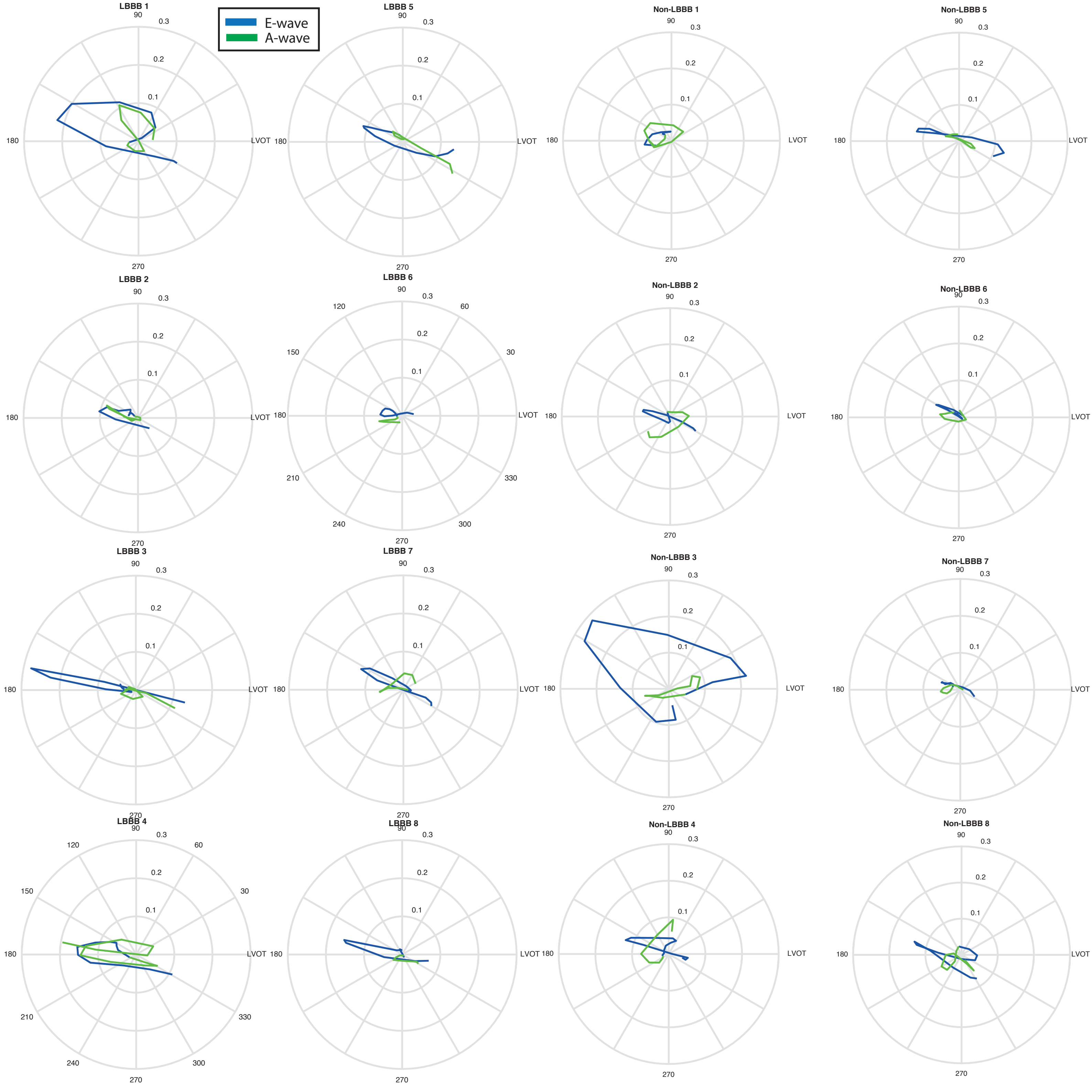

E-wave
A-wave
LBBB 1
LBBB 5
Non-LBBB 1
Non-LBBB 5
LBBB 2
LBBB 6
Non-LBBB 2
Non-LBBB 6
LBBB 3
LBBB 7
Non-LBBB 3
Non-LBBB 7
LBBB 4
LBBB 8
Non-LBBB 4
Non-LBBB 8
90
0.3
0.2
0.1
180
LVOT
270
120
60
150
30
210
330
240
300

Supplementary Figure 2: Hemodynamic forces [N] during diastole projected onto a long-axis (LAx)-plane. The hemodynamic force plots are colored according to diastolic phases, E-wave (blue) and A-wave (green). The x axis is aligned along the apex-to-base axis. E-wave, early diastolic filling; A-wave, late diastolic filling; LA, left atrium; Ao, aorta.

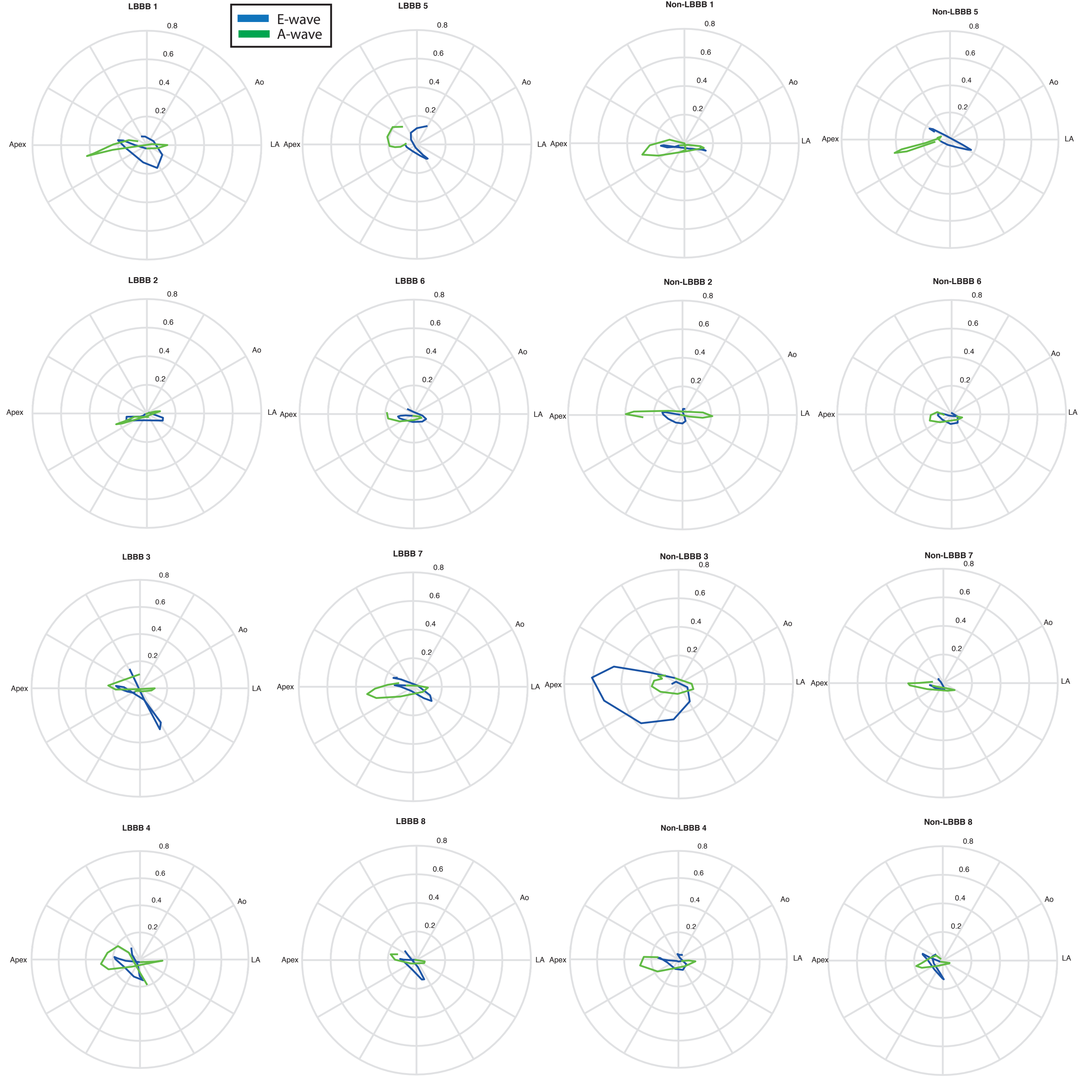
E-wave
A-wave
LBBB 1
LBBB 2
LBBB 3
LBBB 4
LBBB 5
LBBB 6
LBBB 7
LBBB 8
Non-LBBB 1
Non-LBBB 2
Non-LBBB 3
Non-LBBB 4
Non-LBBB 5
Non-LBBB 6
Non-LBBB 7
Non-LBBB 8
0.2
0.4
0.6
0.8
Ao
LA
Apex
